# Improving Maternity Care Where Home Births Are Still the Norm: Establishing Local Birthing Centers in Guatemala That Incorporate Traditional Midwives

**DOI:** 10.9745/GHSP-D-24-00057

**Published:** 2024-10-29

**Authors:** Breanne Lievense, Kaitlin Leach, Nina Modanlo, Ira Stollak, Jaqueline Wallace, Alma Dominguez, Juany Valdez, Mario Valdez, Henry B. Perry

**Affiliations:** a AVAC, New York, NY, USA.; b Optum, SeaTac, WA, USA.; c David Geffen School of Medicine at UCLA, Los Angeles, CA, USA.; d Curamericas Global, Raleigh, NC, USA.; e Independent consultant, Baltimore, MD, USA.; f Curamericas/Guatemala, Calhuitz, Guatemala.; g Health Systems Program, Department of International Health, Johns Hopkins Bloomberg School of Public Health, Baltimore, MD, USA.

## Abstract

More than half of births among Indigenous women in Guatemala are still being attended at home by providers with no formal training. We describe the incorporation of comadronas (traditional midwives) into casas maternas (birthing centers) in the rural highlands of western Guatemala. Although there was initial resistance to the casa, comadronas and clients have become increasingly enthusiastic about them. The casas provide the opportunity for comadronas to continue the cultural traditions of prayers, massages, and other practices that honor the vital spiritual dimension of childbirth close to home in a home-like environment with extended family support while at the same time providing a safer childbirth experience in which complications can be detected by trained personnel at the casa, managed locally, or promptly referred to a higher-level facility. Given the growing acceptance of this innovation in an environment in which geographical, financial, and cultural barriers to deliveries at higher-level facilities lead most women to deliver at home, casas maternas represent a feasible option for reducing the high level of maternal mortality in Guatemala.

This article provides an update on the growing utilization of casas and provides new insights into the role of comadronas as birthing team members and enthusiastic promotors of casas maternas as a preferable alternative to home births. Through the end of 2023, these casas maternas had cared for 4,322 women giving birth. No maternal deaths occurred at a casa, but 4 died after referral.

The Ministry of Health of Guatemala has recently adopted this approach and has begun to implement it in other rural areas where home births still predominate. This approach deserves consideration as a viable and feasible option for reducing maternal mortality throughout the world where home births are still common, while at the same time providing women with respectful and culturally appropriate care.

## Abstract (precis)

Comadronas (traditional midwives) strongly advocate for and participate in attending their clients’ births in local birthing centers in rural Guatemala, where Indigenous women have previously preferred home births because of geographic, sociocultural, and economic barriers to giving birth at a higher-level health facility.

## Teaser

**Key Findings:**

- More than 50% of Indigenous women in Guatemala deliver at home, most often attended by comadronas, traditional midwives who are culturally important, preferred by women over health facilities, and serve as a viable alternative in many parts of the country where health facilities are not close nor easily accessible.
- Community-based and community-supported casas maternas (local birthing centers) provide a feasible alternative for women to give birth in a home-like setting that values comadronas’ involvement and cultural traditions in the birthing process and provide a place where trained health care providers can be present and rapid referral is available should a complication develop.
- Since their introduction in 2009, use of the casas has increased because of the enthusiastic support that comadronas have for them, strong appreciation from clients, and strong support of casa materna staff.

**Key Implication:**

- This approach can be applied not only in Guatemala but also in areas throughout the world where home births predominate and can serve as an “interim” strategy for reducing maternal mortality until higher-level health care facilities become geographically, financially, and culturally accessible and acceptable to these underserved populations.

## INTRODUCTION

More than one-third of births in the least-developed countries are still being attended in homes by informal providers who have no formal training.^1^ This is even true in some lower-middle-income countries such as Guatemala,^2^ where significant regional disparities persist, especially in rural areas, that are masked by national-level statistics. Major barriers still exist throughout the world in giving women universal access to maternity care at facilities staffed by trained personnel where safe deliveries can occur, despite concerted efforts to provide such facilities.^3^^,^^4^ Calls are, therefore, being made for a reconsideration of the role of traditional midwives—“not only as a last resort or stopgap measure when there is no trained or accessible personnel, but as a permanent feature of maternal health systems.”^5^

In 2010, the World Health Organization published updated guidelines for collaborating with communities and families to improve maternal and child health. These guidelines strongly recommend partnering with traditional midwives because of their close ties to communities, cultural knowledge, and social acceptability and standing.^6^ While the value of including traditional midwives has been recognized and these partnerships have been shown to positively impact some maternal and child outcomes, it has been difficult to identify strategies that have led to reductions in maternal mortality.^7^

Multiple strategies have been used to partner with traditional midwives, typically as part of large, complex programs. These strategies can be categorized into 4 broad groups, including programs that (1) improve communication and collaboration between traditional midwives and local health system staff; (2) provide training and education to traditional midwives; (3) use traditional midwives to disseminate health information to the community, and (4) integrate traditional midwives into the health care team.^8^ The implementation of programs that integrate traditional midwives into the health system has led to improvements in the use of skilled birth attendants and increased numbers of antenatal and postnatal visits.^6^

The majority (53.5%) of Indigenous women in Guatemala deliver at home, compared to only 18.6% of non-Indigenous women.^9^ Indigenous women are less inclined to seek maternity care at health facilities operated by the Ministerio de Salud Publica y Asistencia Social (MSPAS, the Guatemalan Ministry of Public Health and Welfare) for a number of reasons, such as the dearth of MSPAS facilities in rural areas, the distance of facilities from the communities where they live (involving hours of driving on treacherous mountain roads), the high cost of transport, the fact that most medical staff do not speak their language, the prohibition of traditional birthing practices at these facilities, and the provision of disrespectful care.^7^^,^^8^^,^^10^^,^^11^ Indigenous women in nearby Chiapas, Mexico, have expressed similar barriers to utilization of health facilities for maternity care.^12^ Private facilities that provide maternity care in rural areas of Guatemala are few and far between and are usually unaffordable to most of the Indigenous population.

Guatemalan women who deliver at home most often rely on the help of a comadrona (traditional midwife).^7^ Comadronas have long played an important role in community health among Indigenous populations in Guatemala, and their calling is regarded as sacred by many.^7^^,^^13^ Besides attending to women during pregnancy and delivery, they are trusted confidantes to women in a male-dominated culture, providing physical, emotional, and spiritual support.

**Figure 1:**
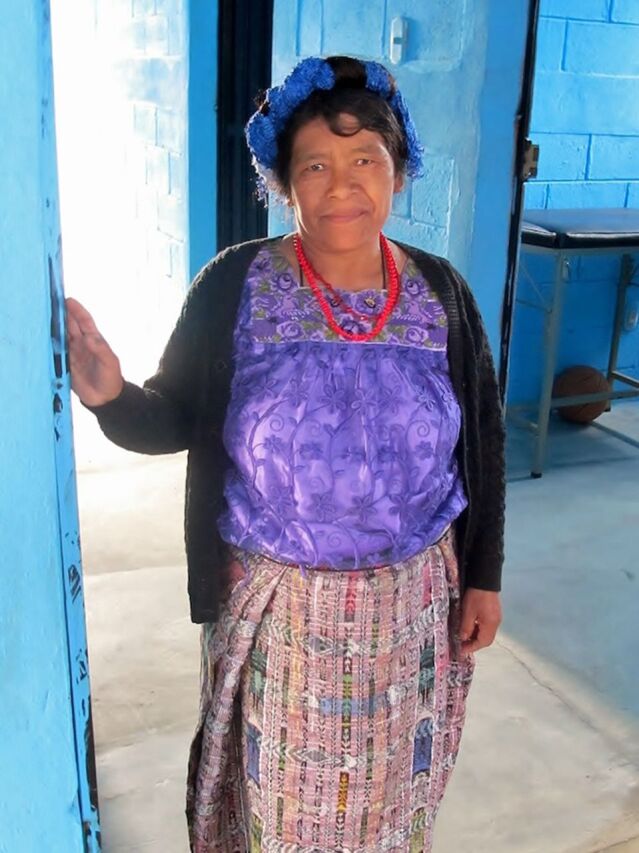
A comadrona at a casa materna, Calhuitz, Huehuetenago, Guatemala. ©2013 Ira Stollak/Curamericas Global

Traditional midwives are also common among both rural and urban marginalized (and often Indigenous) communities throughout Latin America. They are also often referred to as parteras or matrones.^14^ Guatemala has an estimated 8,600 comadronas.^15^ They attend almost one-third (29%) of the country’s births,^9^ and in some rural areas of Guatemala, over 90% of births.^16^ The cultural importance of comadronas, the preference that many women have for them over providers in health facilities, and the lack of viable alternatives in many parts of Guatemala mean that their replacement in the near and medium term is not a realistic option.^15^

Comadronas are authorized by MSPAS to attend home deliveries if they obtain a carnet (documented certification verifying completion of mandatory trainings) and produce a birth certificate for all births they attend.^13^ A number of efforts have been made by health programs in Guatemala to engage with comadronas through training programs to improve their obstetric skills, including formalized trainings sponsored by MSPAS.^16^^,^^17^ These trainings have been faulted for their limited scope, lack of cultural sensitivity, and use of Spanish language rather than the local Maya language.^7^^,^^13^ Furthermore, there is no evidence that this approach has decreased the maternal mortality ratio (MMR) among Indigenous women.^7^^,^^18^ From 2012–2017, MSPAS ran a scholarship training program for Maya-speaking midwives expected to return and work in their communities for 2 years, but it only trained 120 midwives in a 5-year span and did not continue.^19^

Previous efforts of training programs in Guatemala to engage with comadronas to improve their obstetric skills have been faulted for their limited scope, lack of cultural sensitivity, and use of Spanish language rather than the local Maya language.

Special projects have been implemented at referral centers for high-risk pregnancies, including MotherCare’s government-managed casas maternas (local birthing centers) and Project Concern’s casas maternas. However, these casas have not met the ongoing need for “community-friendly” facilities for routine delivery with high-quality, nearby, around-the-clock medical care for mothers and their newborns, nor have they included comadronas as members of the delivery team.^14^

This article provides an update on the growing utilization of casas maternas in the Cuchumatanes mountains in one of the most isolated parts of the Department of Huehuetenango, Guatemala, and highlights the comadronas’ role as members of the birthing team and enthusiastic promotors of casas maternas as a preferable alternative to home births.

We describe the current status of the development of casas maternas in rural Guatemala that include the mother’s comadrona as an integral member of the care team, updating previous descriptions.^10^^,^^20^ We provide findings from interviews with (1) comadronas regarding their experiences of working with the MSPAS, government health facilities, and the casas maternas*;* (2) casa clients regarding their perspectives on the casa and the contribution of comadronas to their birthing experience; (3) casa staff regarding their views of the benefits of including comadronas as members of the birthing team; and (4) observations of a public health graduate student who was present in several of the casas as part of an unrelated research activity.

## MATERNAL AND CHILD HEALTH PROJECT AND ITS CASAS MATERNAS

Since 2002, Curamericas/Guatemala has worked to improve the health of mothers and children in the Department of Huehuetenango, Guatemala. According to official statistics, Huehuetenango has the highest MMR of all the Departments in the country: 215 maternal deaths per 100,000 live births compared to the national level of 113 in 2018.^21^ Between 2011 and 2015, 3 municipalities (districts) in Huehuetenango: San Sebastián Coatán, San Miguel Acatán, and Santa Eulalia, with a combined population of 98,000 people, had one of the highest MMRs in the Western hemisphere: 477 maternal deaths per 100,000 live births.^22^ This is comparable to levels of maternal mortality in many sub-Saharan African countries.

As part of the Maternal and Child Health Project (2011–2015), funded by the U.S. Agency for International Development’s Child Survival and Health Grants Program (hereafter referred to as the Project), Curamericas/Guatemala implemented Project activities in Huehuetenango.

Curamericas/Guatemala, with its U.S.-based supporting partner Curamericas Global, gradually developed and implemented an approach called the Expanded Census-Based, Impact-Oriented Approach (CBIO+) to address inequities in maternal and child health. Previous articles have described CBIO+ and its history,^23^ effectiveness in improving the population coverage of key evidence-based maternal and child health interventions,^24^ childhood nutritional status,^25^ mortality for children aged younger than 5 years and maternal mortality,^22^ and the management of obstetric complications at casas maternas.^20^

### Casas Maternas

In 2009, Curamericas/Guatemala established its first casa materna in collaboration with the community of Calhuitz in the municipality of San Sebastian Coátan. The casa was a feasible alternative to home deliveries, given the inaccessibility of higher-level facility-based care in the area and strong cultural preference of pregnant Indigenous women for involving comadronas in the birthing process. In close collaboration with local Indigenous communities, casas were established in 2013 in Santo Domingo (in San Sebastián Coatán), in 2014 in Tuzlaj Coya (in San Miguel Acatán), and in 2015 in Pett (in Santa Eulalia).

The first casa materna was a feasible alternative to home deliveries, given the inaccessibility of higher-level facility-based care in San Sebastián Coátan and strong cultural preference of pregnant Indigenous women for involving comadronas in the birthing process.

**Figure 2:**
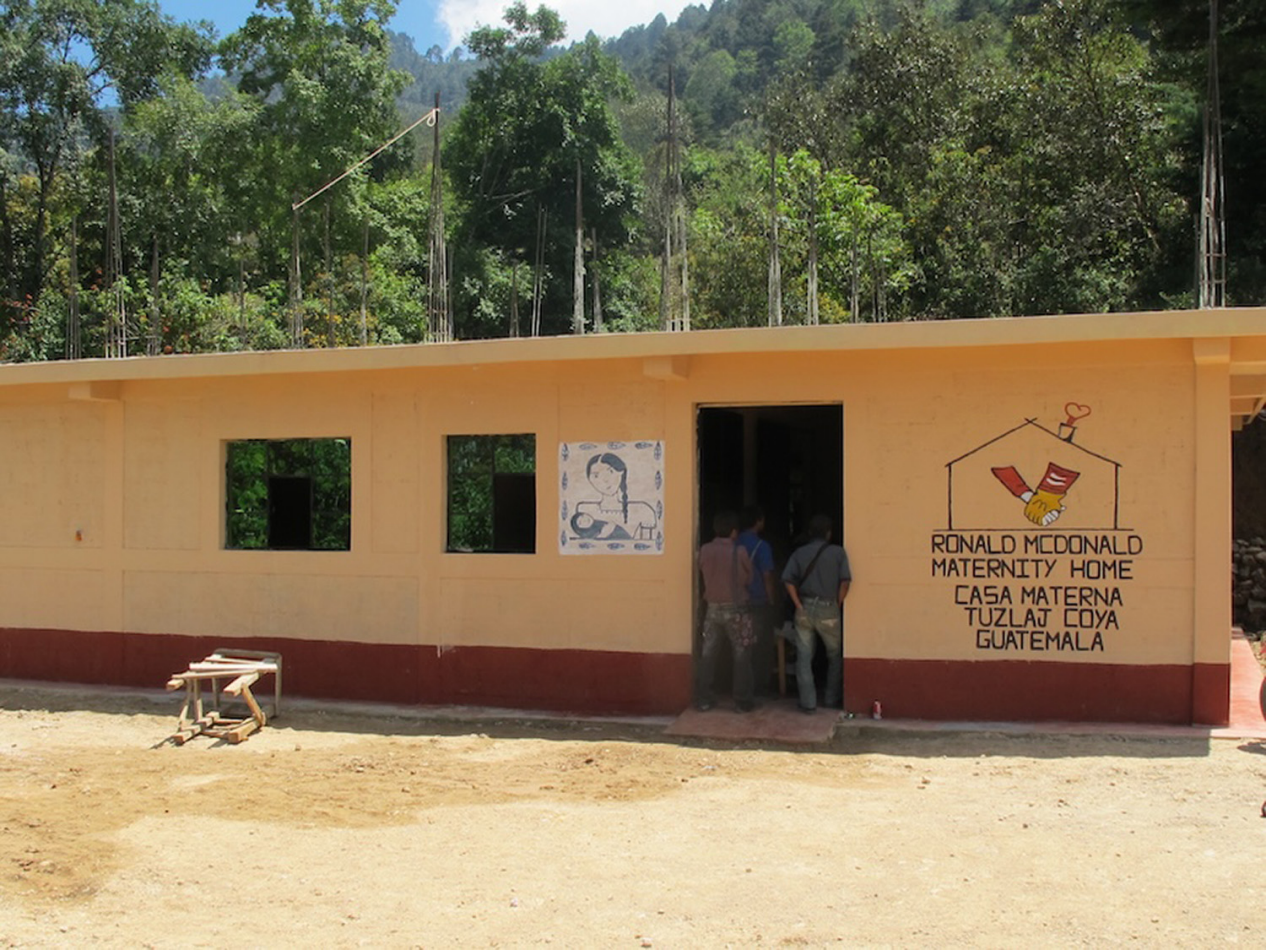
A casa materna, Tuzlaj Coya, Huehuetenango, Guatemala. ©2014 Ira Stollak/Curamericas Global

Detailed descriptions of how these casas function have been reported previously.^10^^,^^20^ In brief, these casas are strategically located within a half-hour drive from the communities they serve. They are constructed and managed with volunteer labor by the surrounding communities (called partner communities). The casas intentionally have an ambience more like an Indigenous home than a clinic, including a traditional kitchen where families can prepare traditional meals and an adjacent sweat lodge (chuj). Hence, the name casa (meaning “home” in Spanish) rather than clinica materna (maternity clinic).

In 2015, the 4 casas were serving a population of 14,846 people. By 2021, the reach of these casas had increased to 20,962 people. As of the end of 2023, there had been 4,322 births at the casas; of these, 720 women (16.7%) were referred to a higher-level facility because of a complication, and 448 (10.4%) women underwent cesarean delivery. No women died at a casa; however, 3 women died on the way to or at a referral facility, and 1 did not accept referral and returned home, where she died.

The casas are staffed 24 hours a day, 7 days a week by local Maya-speaking personnel who are trained in safe birthing practices and closely supervised and continually trained by a graduate registered obstetric nurse. An auxiliary nurse leads the delivery team, which may include other nurses or mujeres de apoyo (support women who function as doulas). Curamericas/Guatemala now provides employment at 2 casas for several midwives with 3 years of formal training in a government-approved university program. These trained midwives are formally called tecnicos universitarios en partería and referred to as parteras (midwives).

The MSPAS adopted the casa materna strategy in its 2023–2032 strategic plan,^26^ and, as a result, there are a growing number of casas now functioning in isolated areas of Guatemala, as will be described later.

## METHODS

In 2023, the Curamericas/Guatemala program staff (MV, AD, and JV), Curamericas Global program staff (IS), and the operations research team (BL, KL, NM, and JW) led by HP consolidated the experience and findings related to the implementation of casas maternas in rural Guatemala. This involved a review of previously unreported data from the 2011–2015 operations research project (other findings of which are reported elsewhere,^27^ as well as more recently collected qualitative data from interviews with comadronas, clients, and casa staff along with the observations of an independent student observer present at casas maternas in the fall of 2022.

As shown in the Table, a series of interviews were conducted over a 10-year span (2013–2023) to understand how comadronas, casa staff, and casa clients view casas and the integration of comadronas into the casa model. These data complement the quantitative data collected routinely on casa utilization and maternal outcomes in the 3 municipalities.^18^

**TABLE.** Data Collection Methodology, Casa Maternas, Three Municipalities, Guatemala

**Year**	**Approach**	**No.**	**Location**
2013	Individual interviews with comadronas		
		20	San Sebastián Coatán
		10	San Miguel Acatán
		6	Santa Eulalia
2015	Group interviews with comadronas		
		18	San Sebastián Coatán
		10	San Miguel Acatán
2018	Group interviews		
		10	San Sebastián Coatán
		10	San Miguel Acatán
2018	Individual interviews with casa maternas clients^a^		
		8	San Sebastian Coatán
		4	San Miguel Acatán
2023	Individual interviews with casa maternas clients		
		6	San Sebastián Coatán
		3	San Miguel Acatán
		3	Santa Eulalia
2023	Written questionnaire for casa maternas staff ^b^		
		10	San Sebastián Coatán
		4	San Miguel Acatán
		4	Santa Eulalia

^a^ Casa clients are those who gave birth at a casa materna.

^b^ Casa staff include nurses, trained midwives, and support women.

In 2013, Curamericas/Guatemala purposively selected 36 comadronas to participate in individual key informant interviews: 20 from the municipality of San Sebastián Coatán, 10 from San Miguel Acatán, and 6 from Santa Eulalia. Interviews were conducted in the local Maya language by educadoras (Curamericas/Guatemala health educators) following an interview script in Spanish developed by a graduate student volunteer. Before conducting the interviews, the educadoras reached consensus on how to best translate the script into the local Maya language. The interviews were audio-recorded and later transcribed into Spanish from the Indigenous Mayan languages (Chuj, Akateko, and Q’anjobal) by the interviewers. Responses were then transcribed from Spanish to English by the graduate student volunteer, who consolidated the findings. Interviews were conducted in secure private locations at the casas.

Structured group interviews were conducted in 2015 with 28 purposively selected comadronas: 18 in San Sebastián Coatán and 10 in San Miguel Acatán. Interviews were conducted at the casa in each municipality (2 group interviews altogether). The interview teams included Spanish- and Chuj- or Akateko-speaking casa nurses and community members led by Spanish-speaking graduate student volunteers (one of whom was a native Spanish speaker), who had translated the interview questions from English into Spanish. The Maya-speaking interviewers translated each interview question from Spanish into Maya and took written notes in Spanish of the informants’ answers given in Maya, translating on the spot. Because the Maya languages spoken by the informants are not written, notes had to be taken in Spanish. With translation assistance from the interviewing team, graduate student volunteers later translated the Spanish notes into English.

To augment and update findings, all comadronas served by the 3 functioning casas in 2018 (Santo Domingo and Calhuitz in San Sebastián Coatán and Tuzlaj in Santa Eulalia) were invited to participate in a group interview. Three group interviews were conducted with 18 comadronas (8 in Tuzlaj, 6 in Santo Domingo, and 4 in Calhuitz). Interviews were conducted in secure private locations at the casas. Interview guides were written in Spanish and translated to Maya languages in real time by bilingual community members conducting the interviews. Interviewees translated and transcribed responses into Spanish in real time, and a graduate student volunteer later translated the responses into English. Also, in 2018, 12 women who had given birth at casas in San Sebastian Coatán and San Miguel Acatán were interviewed by bilingual casa staff. In 2023, 12 casas users (6 in San Sebastián Coatán, 3 in Santa Eulalia, and 3 in San Miguel Acatán) were selected through convenience sampling for individual interviews. Separately, 18 staff members (educators, nurses, and professional midwives) working at different casas shared their opinions via completion of written questions about the contributions of comadronas to deliveries at the casas.

### Ethical Approval

In 2012, we applied for and received approval for our operations research from the Guatemala National Ethics Committee. The research protocol included interviews with comadronas, which were conducted in 2012 and 2015. For each of these interviews, verbal informed consent was obtained, witnessed by a third party, and documented. Respondents were informed that their participation was optional and that there would be no sharing of personally identifying information. Informed consent from study subjects was always obtained in their native Maya language before proceeding with the research activity. The information was provided verbally to the study subjects in the local language and included the purpose of the study, assurance that they were free not to participate and free to end their participation at any moment, and that they would not be denied services if they chose not to participate. They were also given the assurance that their identity would not be released along with the findings. For group interviews, we obtained verbal informed consent witnessed by third parties and documented in the transcripts of those activities. The study was declared exempt from human subjects review by the Institutional Review Board of the Johns Hopkins Bloomberg School of Public Health in 2012 because its faculty member participating in this study (HP) was not responsible for data collection activities and had no access to identifying information about the participants.

The qualitative data collected from clients and staff members in 2018 and 2023 were part of the internal monitoring and evaluation activities of the program and were not a part of the formal operations research carried out between 2012 and 2015. For this reason, formal Institutional Review Board approval was not requested, but appropriate safeguards were taken, the same as those implemented in 2013 and 2015.

Here, we describe the views of those who are embedded in this new integrative approach—comadronas, women receiving maternity care at the casas, and casa staff—to understand the success of the approach and its viability as a sustainable model for safe, dignified maternity care.

## RESULTS

### The Role of Comadronas in Casa Maternas

From 2011–2015, there were an estimated 94 comadronas serving the 3 municipalities (approximately 1 comadrona per 1,000 people). From the outset, the Project considered comadronas to be an integral part of the casa team. Through this model, comadronas continue performing many of their traditional practices for their client before, during, and after the birth but in close collaboration with the formally trained health care staff at the casas.

Through the casa materna model, comadronas continue performing many of their traditional practices for their client before, during, and after the birth while in close collaboration with formally trained health care staff at the casas.

During the prenatal period, the expectant mother contacts the comadrona by cellphone or in person to alert her of the pregnancy and request the comadrona’s services. From that point on, the comadrona visits the mother in her home 3–4 times a month, depending on the preferences of the comadrona and woman. During home visits, the comadrona assesses the health of the mother and baby. The comadrona checks the baby’s positioning, gives massages, offers nutritional advice, and educates the mother on how to recognize danger signs throughout the pregnancy. If the mother plans to give birth in the casa, the comadrona accompanies her to the casa for prenatal check-ups. These comadronas receive monthly refresher training from the Curamericas/Guatemala staff.

Once the mother goes into labor, the comadrona accompanies her to the casa and joins the team there. If a mother in labor comes to the casa without a comadrona, the staff calls one to help. Upon arrival at the casa, the auxiliary nurse collects information from the mother about her health and current symptoms, and the comadrona provides relevant information about the pregnancy up to that point. The auxiliary nurse performs an examination to confirm the baby’s position, listen for a heartbeat, and check for dilation of the cervix. Throughout labor at the casa, the comadrona comforts the mother and performs massages and prayers. The mother is able to wear traditional birthing garb and deliver in the traditional squatting position. When it is time for the mother to begin pushing, the auxiliary nurse(s), support women, and comadrona attend to the mother in the delivery room. Throughout contractions, the comadrona is by the woman’s side and encourages her while the nurse coaches the mother on when to push.

**Figure 3:**
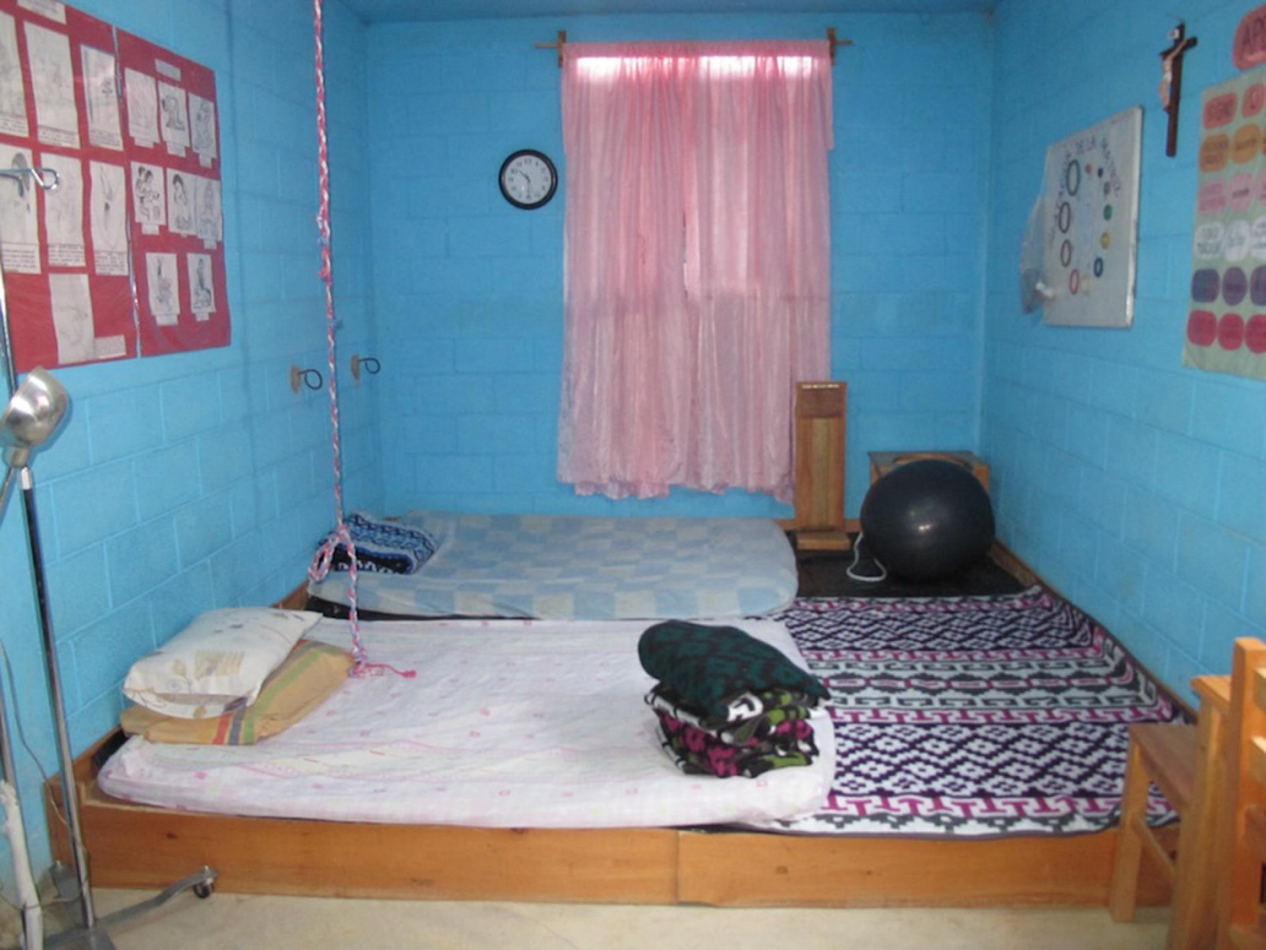
A birthing room in a casa materna, Calhuitz, Huehuetenango, Guatemala. ©2016 Ira Stollak/Curamericas Global

Casa staff implement the accepted best practices of the active management of the third stage of labor, including use of a partograph, uterine massage, uterotonic drugs (oxytocin or misoprostol), and controlled cord traction. At the moment of birth, the delivery team, including the comadrona, works together to perform all the needed newborn care, including drying and wrapping, immediate thermal care, weighing, and facilitating immediate breastfeeding. In addition, the comadrona massages the mother’s lower abdomen to promote uterine contraction (reducing the risk of postpartum hemorrhage) as they await the delivery of the placenta. The support staff collect the placenta as it is expelled while the comadrona continues to massage the abdomen. During this time, the comadrona also offers special prayers. Meanwhile, the extended family is present in the casa kitchen, where ritual meals and teas are prepared on a traditional stove, ritual prayers are said, and songs are sung. After the delivery, this food is shared with the mother.

Once the baby is cleaned, weighed, and wrapped, the comadrona and support staff help the mother breastfeed. The comadrona comforts the mother and her baby. Several hours postpartum, the comadrona helps the mother into the chuj (a traditional sweat lodge located in the casa patio). The comadrona stays with the mother in the casa until she and the baby are released 12 hours after the birth if there are no complications, according to MSPAS policy followed at the casas. For 3 days after the birth, the comadrona tends to the mother and baby at the mother’s home, checking for signs of fever, headache, or other signs of postpartum complications. Although the comadronas were not asked specifically about the fee they charged, the Project staff stated that they usually charged between Q500 to Q1,200 (Guatemalan quetzals, equivalent to US$70–US$170) for the services they provide for each pregnancy. Sometimes, payment in kind was provided, such as with food.

An important aspect of the comadronas’ role at the casas is their responsibility to attend trainings. Long before the casas became operational, Curamericas/Guatemala nurses were offering monthly trainings to comadronas in the local Maya language in coordination with the MSPAS, teaching them how to promptly detect and refer women with obstetric emergencies and implement essential practices for care of the newborn, all according to a Home-Based Life-Saving Skills program developed by the American College of Nurse-Midwives.^28^ Once each casa became operational in a locality, these trainings continued but with an emphasis on birth at the casa rather than in the home. These trainings provided the opportunity for comadronas to work with the casa staff, foster an awareness among them of the benefits that a delivery at the casa can provide, and facilitate incorporation of comadronas into the casa team.

In addition to prenatal checks provided by the comadronas, women also receive prenatal checks following MSPAS protocols at the casas done by the auxiliary nurses, as well as prenatal and postpartum home visits by educadoras, who provide pregnancy education and counseling in the local Maya language. Thus, the comadronas are not merely an “add-on” but rather are integrated into and an essential part of a holistic local maternal health care experience.

The introduction of casas has made it possible to honor the cultural tradition of a strong role for comadronas while simultaneously providing a much safer birthing environment. Casas integrate traditional cultural practices with modern medical practice by allowing women to wear traditional birthing garb; encouraging deliveries in the traditional squatting posture; and enabling the preparation of birth ceremony foods in the casa kitchen, the mother’s consumption of traditional herbs, the use of the traditional chuj following birth, and communication in the local language. Furthermore, comadronas participate in the delivery process in a way that respects their traditional role of offering prayers, comfort, and massages.

### Reflections From Comadronas

#### Interactions With the Ministerio de Salud Publica y Asistencia Social and Government Health Facilities

Comadronas interviewed in 2013 and 2015 shared similar sentiments regarding their role in maternity care. In general, most comadronas had little or no direct interaction with the MSPAS and were not familiar with its role. However, a few mentioned that they had attended MSPAS trainings where they had received some supplies (e.g., towels, scissors, clamps) and maternal vitamins. Many comadronas expressed that the MSPAS had made it difficult for them to practice, and they believed the MSPAS did not value their work. Comadronas were frustrated with the national system requiring them to have a carnet to practice as midwives. The government system also required them to produce birth certificates for the babies they delivered —a problematic requirement because comadronas were not capable of producing one on their own. Comadronas were also expected to attend trainings at locations that were difficult to access.

Several comadronas reported negative experiences while accompanying patients to a government hospital or other government facility. Some women had to wait unattended for lengthy periods of time. While there, comadronas and family members were sometimes separated from the client and treated dismissively and disrespectfully by health facility staff.

*Whenever I have gone to a [government] health center, they have always received me reluctantly – if it’s necessary for me to enter, they’ll let me enter. But the staff there never see me as a vital person in the culture or as a contributor to the delivery.* —Comadrona

#### Partnership With Casas

In contrast with their interactions with MSPAS, comadronas expressed several benefits of working with casas. For example, comadronas appreciated that casa staff could provide birth certificates for their clients. Comadronas also reacted positively to monthly trainings provided by casa staff. They said that trainings, delivered in the local Maya language by bilingual casa staff at a readily accessible location, increased their knowledge about pregnancy, labor, and delivery and provided the opportunity for increased communication with and recognition from casa staff. Comadronas acknowledged their responsibility to learn and apply their new knowledge and skills, and they reported that the trainings also provided a clear understanding of their limits.

Comadronas expressed several benefits of working with casas, including monthly training delivered in the local Maya language by bilingual casa staff to increase their knowledge about pregnancy, labor, and delivery.

*I am very thankful for the educational talks and trainings. Because of them we don’t have insecurities and we have much more confidence when seeing the pregnant women.* —Comadrona

Comadronas stated that their role had changed significantly following the establishment of the casas. Most notably, comadronas began regularly encouraging women to give birth in the casas rather than at home. Some comadronas even stated that they told clients they would work with them only if they agreed to give birth at a casa. Comadronas also acknowledged the collaborative nature of attending births at casas as a major shift in their role. Many comments were made by comadronas about the difficulty of managing a home birth alone with no other help and the enormous benefits of working with a team at the casa so that the numerous tasks that need to be performed simultaneously around the time of birth could be carried out by the team instead of by 1 person. Comadronas agreed that this shared responsibility had relieved them of a great deal of pressure, and they felt better knowing that if a complication arose, they would have additional support and would not have to assume the entire blame themselves if something were to go wrong. Consequently, comadronas began experiencing a new sense of confidence in their work and in the quality of care provided to clients.

*I give thanks to God that the casa came because I always bring my pregnant women there and they help a lot. There were times before when the women died because there wasn’t help from other people. We were alone and in charge. Then when the casa came, it helped a lot and it is for the well-being of everyone, not just of some but everyone.* —Comadrona

*The difference is that during a home birth, the Comadrona is in charge of everything – taking care of the baby and taking care of the mother as well. But in the casa, I attend to the woman while others attend to the baby, and they also attend to the other parts of the delivery, such as waiting for the placenta. But at the time of a delivery at the home of the mother, there is no one to support me.* —Comadrona

*In the casa materna, when a woman gives birth there, help is available. I need their help as much as they need mine. The difference is that if it were in the home, I would be alone there. There are times when I need help, but I am alone, and I have to fight so that the woman gives birth. In the casa materna there is more help.* —Comadrona

Comadronas shared positive remarks about the casas staff, noting a sense of teamwork and partnership when working with them. Comadronas said that casa staff made them feel welcome, supported, and valued as an integral part of the delivery team. At the casas, comadronas and casa staff were learning from each other and forming a team in which every member was important.

Comadronas agreed that a casa delivery was preferable to a home delivery. Reasons for this included (1) the casa staff were highly skilled, (2) the casas were clean (most local homes had dirt floors, making cleanliness a challenge), (3) the casa had medications and sterile materials that were ready for use at the time of delivery, and (4) the casas could promptly refer a mother to the hospital if a complication arises that cannot be managed in the casa. Comadronas felt that the casa staff had been effective in integrating them into the care of women during delivery at the casas.

*At the casa materna, everyone receives me and other comadronas with open arms; there is no discrimination [in contrast to how we are received at an MSPAS facility].* —Comadrona

Group interviews in 2018 revealed that comadronas held positive perceptions of their partnership with the casas. Comadronas reported being completely integrated into the casas care team and throughout the birthing process. They felt supported and valued by casas nurses and staff, especially due to the respect shown toward the traditional cultural practices of the comadronas and the women they served.

*The casa materna takes into account our work; they value us and invite us to support them, and they treat me as a leader within the team.* —Comadrona

Just as in the interviews conducted in 2013 and 2015, comadronas interviewed in 2018 felt confident working with the casas and encouraged women to deliver there because of the high-quality services and medications available there.

*The quality of services offered by the maternity home is very important for the well-being of the entire community and partner communities as well.* —Comadrona

#### Barriers to Casa Utilization

Comadronas interviewed in 2013 and 2015 cited several barriers to casa utilization. Some comadronas stated they initially feared using casas. Others viewed them as birthing places only for complicated deliveries, which prevented them from encouraging clients to use casa services for routine pregnancies. As exposure to and familiarity with casas and staff increased, comadronas said their fears were alleviated and began referring all clients to a casa. The barriers noted earlier were not observed in 2018.

However, a more persistent barrier across interviews was that comadronas did not always participate in a family’s decision regarding where the birth would take place. Comadronas said they spent a lot of time trying to convince families to allow the mother to give birth at a casa but sometimes encountered resistance because of the families’ traditional beliefs about the necessity of a home birth. Distance was another persisting barrier, especially for those women who lived further from casas. Comadronas in 2013 and 2015 interviews also suggested casas could be improved with more staff, additional medicines, and an ultrasound machine.

### Reflections from Casa Materna Clients Who Gave Birth in a Casa

#### Acceptance of Casas

The casas and their integration of comadronas have also been viewed positively by women receiving services. All women interviewed in 2018 stated they would recommend the casa to their friends. The women said their opinions and wishes were valued and listened to while they were cared for in the casa. Specifically, they expressed gratitude for the opportunity they had to practice cultural traditions at the casa during childbirth and to include their comadrona. All the clients were strongly in favor of giving comadronas a role in their delivery at the casa.

Women who gave birth at a casa expressed gratitude for the opportunity to practice cultural traditions at the casa during childbirth and include their comadrona.

*They [the casa staff] respected my beliefs, they respected my traditional clothing, and they respected the presence of my comadrona with me, enabling her to give me traditional massages and to ask for forgiveness before giving birth.* —Client

*There [in the casa] it gives us the sense of feeling at home, with confidence and without fear.* —Client

*The [casa] staff have earned the trust of the people.* —Client

The women noted the importance of the casas for their communities, attributing the reduction in maternal mortality and post-childbirth complications to the casas.

*Thanks to the casa, there have no longer been maternal and neonatal deaths, and it is for that reason that I trust the casa and its staff.* —Client

#### The Vital Role of Comadronas

Clients interviewed in 2023 mentioned that their comadrona provided them “confidence,” “emotional support,” and “encouragement” through their presence because they knew the mother well, had often attended their previous birth, and had knowledge inherited through many generations. Clients said that they “trusted” their comadrona. Typical statements about the comadronas included the following.

*With my comadrona I feel safer because she knows me and has already helped me with my first children.* —Client

*They make us feel safe at the time of our delivery.* —Client

*Next to my comadrona I feel at home.* —Client

*They support us. We consider them as a mother, someone we trust more.* —Client

*They are like our second mother – they give us advice and confidence.* —Client

*They have a lot of knowledge and experience.* —Client

*We would feel alone without them.* —Client

*She supports me by doing my prayers with me.* —Client

*They have a lot of knowledge inherited from their grandmothers from generation to generation.* —Client

Clients interviewed in 2023 also mentioned that their comadrona provided them with physical comfort by massaging their abdomen and back during contractions and by offering medicinal plants and natural medicines.

*The comadrona is always there supporting. During contractions she performs massages to help with the development of labor.* —Client

*The comadrona was giving me massages during the contractions and together with the nurses they helped me to do exercises to facilitate my delivery.* —Client

*She provided me with tea to have enough milk to breastfeed my baby.* —Client

### Reflections From Casa Staff

All casa staff members interviewed in 2023 were uniformly and enthusiastically affirmative regarding the value of including comadronas. They described the comadrona’s role at the time of delivery of their client.

- Encouraged women to come to the casa for prenatal check-ups and delivery.

*Comadronas are leaders in the communities, so they are a great support to the casas in encouraging mothers to give birth there.* —Casa staff

*They are people with a lot of wisdom.* —Casa staff

*Mothers and families have great respect for them.* —Casa staff

*The mother does what the comadrona tells her to do.* —Casa staff

- Served as a link between the community and the casa.
- Provided the mother with emotional and psychological support, giving the mother confidence and trust.

*She makes the mother feel more confident and gives her more security.* —Casa staff

*They know the reality of the patient.* —Casa staff

*She helps the mother to feel safe and confident.* —Casa staff

- Helped to build trust between the mother and the casa staff.
- Ensured dignified maternity care.

*They help to provide a culturally appropriate, humanized, and respectful childbirth.* —Casa staff

- Provided the mother with massages (rubbing her head, abdomen/uterus, and back), breathing exercises during labor, support for pushing at the time of delivery, and uterine massage after delivery of the placenta.
- Assisted the mother in getting into a comfortable position.
- Assisted with language translation (needed only occasionally).
- Gave natural medicines and tea for pain control and relaxation.
- Took charge of the newborn.

*Comadronas take charge of providing care to the newborn, such as tying the umbilical cord and wrapping them in a warm blanket.* —Casa staff

- Assisted the rest of the staff and promoting teamwork among those providing care.
- Provided follow-up care at home following delivery.
- Provided guidance to women in family planning and sex education.

### Reflections From an Independent Student Observer

During the fall of 2022, a Johns Hopkins Bloomberg School of Public Health master’s student, Rose Greer, spent 2 months collecting data for her capstone project on respectful maternity care, during which time she was inside several of the casas observing the activities. She provided the following comments through personal communication (April 6, 2024).

*From my perspective, it seemed that the comadronas were very well integrated into the care team at the casas. The comadronas would often attend to the non-medical needs of the patient, such as preparing food and beverages and performing pain management exercises. Though this was never directly expressed to me, this division of labor seemed beneficial, as it allowed the casa nurses to focus on preparing for the birth with the confidence that the mother was well taken care of by the comadrona.*

*On occasion, the casa staff would share their thoughts on comadronas who utilized the casa for birthing or other services. I recall once when a comadrona brought a mother and newborn to the casa for birth registration (following a home birth) and a nurse at the casa expressed disapproval that the comadrona had attended to the birth in the home rather than bringing the patient to the casa.*

## DISCUSSION

Our data indicate that delivery at a casa materna is associated with an eight-fold reduction in maternal mortality relative to home delivery. The experience of implementing casas maternas where women were able to bring their comadrona with them to participate in the delivery demonstrates that they are gaining acceptance as a place for giving birth that is convenient, affordable, and safer than a home birth while at the same providing a humane experience that affirms deeply held cultural values and traditions essential to their dignity and identity as Indigenous persons.

Our data indicate that delivery at a casa materna is associated with an eight-fold reduction in maternal mortality relative to home delivery.

The interviews with comadronas, recent clients, and casa staff all confirmed these findings, and an independent observer testified to the harmonious teamwork that developed between the formally trained casa staff and the comadronas who came to the casa with their client when she was in labor. The Project has successfully integrated comadronas into its maternal health services, giving them an alternative way to continue their work in sharp contrast to the marginalization that many comadronas had experienced in their interactions at MSPAS health facilities.

Comadronas view their partnership with casas as a positive change. They have become a member of a strong team dedicated to providing women with a safe, respectful, and culturally appropriate delivery and were grateful that the responsibility for the care of the mother and newborn no longer lay completely in their hands. Comadronas have gained renewed confidence and pride in their work because they could provide their clients with culturally appropriate care in a setting that could respond more effectively if and when complications occurred. They also valued the ongoing and readily accessible trainings they received at casas.

Comadronas have become important allies in the effort to adopt new practices without totally abandoning the old ones. For example, women who delivered in the casas often cited the encouragement of their comadrona as the main factor driving their decision to deliver there.^11^ Furthermore, the casa model not only enabled comadronas to continue in their traditional role but also enabled them to continue being paid by families for their work. Thus, casas did not threaten the legitimacy of comadronas or their income but rather provided them with the opportunity to continue their traditional role in an environment in which improved quality of care could be provided.

Despite overwhelming positive views of casas expressed by comadronas and clients, casa utilization is not without its challenges. Long-standing cultural traditions and beliefs are slow to change, particularly in the 3 municipalities where there is limited interaction with the broader society. Even after comadronas became champions for casas, they still had challenges in persuading some families to use the casa. Cultural attitudes and perceptions that favored home births presented a major barrier to access and utilization of services provided by casas, even when comadronas strongly encouraged utilization. In addition, long travel times presented as a barrier to casa utilization. Each casa is located 8 km–10 km from some of the communities it serves, but some women may still view this distance as too far to travel while in labor compared to the convenience of a home birth. Increasing casa usage will require changing traditional attitudes and perceptions, and this will require community-wide efforts.

Regarding comadrona suggestions in 2013 and 2015 for additional medicines and ultrasound machines, all casas are now equipped with a botiquin (medicine chest) of basic drugs, including oxytocin for the management of the third stage of labor (delivery of the placenta following the birth of the baby), and an ultrasound machine.

Interviews with casa clients revealed strong community support for the casas. The women deeply appreciated the ability to work with a comadrona and practice cultural traditions while giving birth at a casa, which allowed them to feel at home and facilitated trust of casa staff. These interviews also underscored the vital role comadronas play in maternity care in the 3 municipalities. Women noted reliance on comadronas for physical and emotional support throughout pregnancy, delivery, and the postpartum period. From the perspective of casa staff, comadronas created a critical link between casas and communities. Because comadronas hold important influence in communities, they served the casa team by encouraging women to give birth in a casa and providing maternal education, which may not have been as well received from casa staff alone. Furthermore, comadronas supported casa staff at the time of delivery, providing physical and emotional support to the mother in a way that allowed casas to provide dignified care.

Because comadronas hold important influence in communities, they served the casa team by encouraging women to give birth in a casa and providing maternal education, which may not have been as well received from casa staff alone.

The Project is just one of many efforts underway to affirm the important cultural role that comadronas perform while at the same time integrating them into the health system in a way that can improve the quality of maternal and neonatal care for Indigenous women in Guatemala. For example, the Maya Health Alliance works with comadronas in 5 departments of Guatemala to support them in their efforts to improve the quality of the care they provide by giving them mHealth tools that can assist them in the detection of complications in need of referral,^17^^,^^29^ fostering closer collaboration with hospitals and transport providers,^30^ and incorporating local obstetrical care navigators who can accompany patients who have been referred to a hospital, thereby reducing some of the barriers to acceptance of referral when complications arise.^30^ The Asociación de las Comadronas del Area Mam provides health care, transportation, and referrals through their birth center and mobile clinics and holds continuing education clinics for local comadronas and health workers.^31^ The Departmental Coordinator of Traditional Comadronas of Quetzaltenango supports a network of 800 comadronas in the Department of Quetzaltenango by providing equipment and training to deliver safer, more sophisticated care.^32^

As a result of all of these efforts and experiences, the MSPAS has now included the construction and operationalization of casas maternas in priority areas of the country in collaboration with communities as an important part of its national strategy for reducing maternal mortality nationally over the next decade (2022–2032).^26^ As of July 2024, there are 9 casas maternas operated by MSPAS (5 in the Department of San Marcos, 3 in the Department of Barillas, and 1 in the Department of Quiché). Most of these have started functioning since the release of the new 2022–2032 MSPAS strategic plan. In addition, there are approximately 6 casas maternas operated by other nongovernmental entities throughout Guatemala that incorporate comadronas into their birthing teams. Comadronas are now experiencing a renaissance of recognition and dignity.^32^^,^^33^

### Experiences Beyond Guatemala With Local Community Birthing Centers

Similar but not identical approaches have been implemented elsewhere. Gabrysch and colleagues^34^ developed a nongovernmental organization-facilitated culturally friendly birthing center in Ayacucho, Peru, for an Indigenous population where the percentage of births at the center increased from 6% to 83% over an 8-year period. However, there is no indication that traditional midwives played any role in the care of the mother during the birthing process. One report from Pakistan^35^ describes the integration of traditional midwives into the health care team, with traditional midwives providing physical and cultural support to the birthing woman and assisting with translation, similar to the Curamericas/Guatemala Project. However, this program integrated traditional midwives into existing health centers and hospitals, not community-supported local birthing centers.

The literature on community birthing centers in Latin America comparable to casas maternas is limited, but there are 2 published examples in which similar approaches have been tried unsuccessfully.^36^^,^^37^ In both, traditional midwives were not integrated into the maternity care provided in the community birthing center. In addition, community engagement and community ownership were absent in these 2 cases, suggesting that these factors—which were critical for constructing and managing the Guatemala casas—are particularly important for explaining the success of the casas in the Department of Huehuetenango.

While there is extensive published literature on birthing centers for low-risk deliveries staffed by formally trained midwives in high-income settings, we have been able to identify only 1 report of a community-based maternity center that has integrated traditional midwives into a community birthing center in a low-income setting.^38^ In that case, traditional midwives living in urban slums of Bangladesh were recruited and trained to provide maternity care at birth huts that served areas with approximately 10,000 people. Two traditional birthing attendants staffed each birthing hut so that 1 person was always on duty 24 hours a day, 7 days a week. A referral system was well-developed and available for women who developed complications.

### Limitations

We recognize that the methods used for collecting the qualitative data in this article do not meet optimum quality standards. However, the number and types of respondents included in our qualitative data collection procedures provide a counterbalance. Altogether, we interviewed 82 comadronas, 24 casa clients, and 18 casa staff members.

Another possible limitation of our data is the loss of meaning or content due to language challenges during interviews. Because several different people translated between Mayan, Spanish and English languages, varying levels of language proficiency held by translators may have resulted in imperfections in the meaning of questions or responses. However, the staff were quite experienced with translating since it was part of their day-to-day work providing services and education in the casas and communities, and most staff were native speakers of the Maya languages. Moreover, comadronas and casa clients may have had a conscious or unconscious desire to please interviewers who were affiliated with the Project and the casas, possibly leading to a favorable response bias.

## CONCLUSIONS

In a broader context, the Indigenous population of Guatemala has suffered centuries of discrimination, marginalization, exploitation, and, at times, outright genocide. The formal health system also reflects this marginalization and contributes significantly to the poor health outcomes experienced by Indigenous Guatemalan women and children. The casa approach, with its foundation in the empowerment of and partnership with Indigenous communities, including their comadronas, represents an important step away from this tragic marginalization.

Furthermore, incorporating traditional midwives into local, readily accessible, community-based, and community-supported birthing centers that have more highly trained (but still low-level) staff, follow protocols for safe delivery, and have a system for prompt transport of women with complications represents a useful “middle way” in other geographical areas around the world where home births are still the norm and higher-level health facilities are not close nor readily accessible (and may never be due to profound economic, political, and geographic constraints). The casa materna approach represents a potentially feasible and effective strategy for reducing the unnecessarily high levels of maternal, perinatal, and neonatal mortality while at the same time providing respectful care that honors these cultural traditions and integrates communities and their rich social capital. To accelerate the decline of maternal mortality and facilitate the provision of essential newborn care in rural areas, the development of locally available birthing centers similar to the casas developed in rural Guatemala and the integration of the functions of traditional midwives into the services provided at these centers can not only be a useful strategy but also serve as a new paradigm for the creation of more effective and just local health systems that engage communities as active partners. The adoption of the casa materna strategy by the MSPAS for its 2023–2032 strategic plan^26^ to reduce maternal mortality reflects the government’s recognition of the value of the pioneering work of Curamericas/Guatemala.

While we agree with those who argue that all births should eventually take place in or near hospitals that are capable of handling obstetric and newborn emergencies,^39^ this will not be feasible for the near or medium term in many settings such as the Western Highlands of Guatemala and other similar areas around the world with topography that hinders transport, dispersed populations, and under-resourced health systems. This approach will also have important limitations in areas of political conflict, violence, and humanitarian disasters where fully functioning higher-level health systems are no longer available. Thus, in the near term, less than ideal approaches (at least from the quality of medical care perspective) that are nonetheless feasible and that are clearly an improvement over the status quo are needed for a major segment of the world’s population. And localized context-specific birthing centers may, in fact, represent an ideal approach if maternal mortality there can be shown to be as low as the maternal mortality in higher-level facilities.

The 2023 World Health Organization report on global maternal mortality^40^ highlights a disappointing lack of progress between 2010 and 2020, with the global level remaining at 223 maternal deaths per 100,000 live births, still far above the goal of 70 set to be achieved by 2030 as part of the Sustainable Development Goals. The report calls for “substantial shifts in focus and investment” to meet the unprecedented challenge of achieving an annual reduction of 11.6% in maternal mortality globally that will be required to meet this Sustainable Development Goal. Elsewhere, we have shown that the Project, over its 4 years of implementation (2011–2015), was able to reduce maternal mortality in its service area by 59%.^22^ The development of local birthing centers that integrate traditional midwives into their functioning holds promise as an approach for accelerating the decline in maternal mortality in the many areas around the world where home births predominate.

## Notes

**Peer Reviewed**

**First Published Online:** September 19, 2024.
